# N-Terminal Region of the Catalytic Domain of Human *N*-Myristoyltransferase 1 Acts as an Inhibitory Module

**DOI:** 10.1371/journal.pone.0127661

**Published:** 2015-05-22

**Authors:** Sujeet Kumar, Rajendra K. Sharma

**Affiliations:** Department of Pathology and Laboratory Medicine, Cancer Cluster, College of Medicine, University of Saskatchewan, Saskatoon, Saskatchewan, Canada; University of Oldenburg, GERMANY

## Abstract

*N*-myristoyltransferase (NMT) plays critical roles in the modulation of various signaling molecules, however, the regulation of this enzyme in diverse cellular states remains poorly understood. We provide experimental evidence to show for the first time that for the isoform 1 of human NMT (hNMT1), the regulatory roles extend into the catalytic core. In our present study, we expressed, purified, and characterized a truncation mutant devoid of 28 N-terminal amino acids from the catalytic module (Δ28-hNMT1s) and compared its properties to the full-length catalytic domain of hNMT1. The deletion of the N-terminal peptide had no effect on the enzyme stability. Our findings suggest that the N-terminal region in the catalytic module of hNMT1 functions serves as a regulatory control element. The observations of an ~3 fold increase in enzymatic efficiency following removal of the N-terminal peptide of hNMT1s indicates that N-terminal amino acids acts as an inhibitory segment and negatively regulate the enzyme activity. Our findings that the N-terminal region confers control over activity, taken together with the earlier observations that the N-terminal of hNMT1 is differentially processed in diverse cellular states, suggests that the proteolytic processing of the peptide segment containing the inhibitory region provides a molecular mechanism for physiological up-regulation of myristoyltransferase activity.

## Introduction

*N*-myristoyltransferase (NMT; EC 2.3.1.97) is a central switch in cellular signaling pathways, which catalyzes the covalent transfer of tetradecanoate (C14:0) to the N-terminal glycine in either a co- or post transnational manner [1–5]. The myristate (C14:0) is linked to N-terminal glycine by stable amide bond and the half-life of myristoyl moiety on a protein is thus equivalent to the half-life of the polypeptide chain backbone [4]. Myristoylation increases lipophilicity and thus promotes its substrate proteins to achieve their specific cellular roles [6–8]. The pivotal role of NMT’s in ensuing cell survival makes them a pharmacological target in a number of diseases, most notably cancer and several parasitic and infectious diseases [9–16]. Aberrant myristoylation has also been linked to the spontaneous development of a rare genetic disorder resulting in reduced growth, facial dysmorphism, cognitive deficits and malignancies. The disease phenotype has been attributed to the spurious myristoylation of the SHOC2 protein harboring S2G mutation [17].

There are two isoforms of NMTs (NMT1 and NMT2) in higher eukaryotes which are encoded by different genes and have different specificities [18]. Among the two isoforms, NMT1 knockdown is shown to inhibit tumor growth *in vivo* and is thus a validated direct target in cancer [19, 20]. We have recently shown that NMT1 is essential for the development of mouse embryo and its knockdown results in defective myelopoiesis in mouse [21, 22]. Also, previous observations have shown that among the two NMTs in humans, NMT1, but not NMT2 is, processed to multiple isoforms [18]. It is not fully understood how these *in vivo* regulations are conferred. The polypeptide segment encompassing the 416 amino acids from residue 81–496 defines the catalytic module of hNMT1 [23]. Earlier reports have established that the first 80 amino acids of NMT1 are involved in ribosomal targeting in consistence with its function for co-translational myristoylation [24]. What remains unresolved is whether the defined catalytic module of hNMT1 [designated as hNMT1s; [25]] serves the minimal catalytic core or it encompasses domains, which have non-catalytic functions. This prompted us to reinvestigate the catalytic domain of hNMT1 and rigorously define its boundaries. Given the importance of hNMT1 in pathogenic states it is important to assign the catalytic and regulatory roles to different regions of the enzyme and to delineate the minimal functional domain to serve the requirement for the screening large chemical libraries for identification of lead compounds with enhanced binding affinities.

In this report, we therefore addressed two primary questions: Firstly, does the defined catalytic module, hNMT1s, serve the minimal catalytic core, and secondly, in comparison to the catalytic domain reflected by the kinetoplastid parasites, how enzymatic properties of hNMT1s are modulated by the N-terminal extension. We have focused on the full-length catalytic domain (i.e hNMT1s) and in its comparison, characterized the truncation mutant devoid of 28 amino acids at the N-terminal tail (hereafter referred as Δ28-hNMT1s). Employing the single vector system for myristoylation [26], we first established the functionality of Δ28-hNMT1s in complementation assays system in *E*. *coli*. An enzymological approach was undertaken to determine the extent to which this atypical extension affects the catalysis. Complete removal of the 28-amino acid long N-terminal tail region results in a gain of function and enhanced kinetic properties without compromising the stability of the molecule. Our findings suggest that this region controls enzyme functionality by regulating the peptide substrate affinity to the enzyme active site. Sequence variations among the N-terminus region among discrete NMTs thus may serve as a regulatory mechanism for the physiological regulation of NMT activity.

## Materials and Methods

### Materials

Standard chemicals were purchased from Sigma, polymerases were obtained from MBI-Fermentas and restriction enzymes and ligases were from New England Biolabs (NEB). *E*. *coli* strain NEB 5-α (NEB) was used for amplification of plasmids. Oligonucleotides used in the generation of expression constructs, myristic acid azide (12-Azidododecanoic Acid; Az-Myr) and Alexa Fluor 488 DIBO Alkyne (Labeling reagent; LR) were from Invitrogen. Culture media, Antibiotics, buffer salts and isopropyl β-D-1-thiogalactopyranoside (IPTG) were purchased from EMD chemicals. Protein expressions were performed in *E*. *coli* strains BL21 (DE3) or Rosetta 2(DE3) as indicated. All purifications were performed on resins and columns from GE Healthcare. Myristoyl coenzyme A (MYA) lithium salt and 7-diethylamino-3-(4-maleimidophenyl)-4-methylcoumarin (CPM) for use in enzymatic assays was obtained from Sigma while the peptide substrate was custom synthesized from the Institute for Biomolecular Design (University of Alberta) at a purity scale of >95%. The peptide substrate employed in enzymatic assays corresponds to the N-terminal sequence of the pp60^src^ (amino acids 2–9; GSNKSKPK). Plasmids pETDuet-1Δ6His_Nef and pETDuet-1Δ6His_hNMT_Nef were obtained from Dr. Dieter Willbold (Jülich, Germany) and has been described elsewhere [26].

### Sequence and structural analysis of the N-terminal region of NMT

NMT coding sequences from diverse organisms were retrieved from the Uniprot database [27]. The divergent N-terminus from hNMT1s was compared to the corresponding regions of orthologous NMTs from *Trypanosoma brucei* (Q388H8), *Trypanosoma cruzi* (Q4E2V2), *Leishmania donovani* (D0AB09), *Caenorhabditis elegans* (G8JY05), *Plasmodium vivax* (A5K1A2), *Glycine max* (I1KUQ9), *Oryza sativa* (Q8LR54), *Arabidopsis thaliana* (Q9LTR9), *Phytophthora infestans* (D0N9Y9), *Candida albicans* (P30418) and *Saccharomyces cerevisiae* (P14743). The numbers in brackets correspond to the Uniprot accession numbers of the NMTs from respective organisms. The sequence conservation at the N-terminal region among representative NMT proteins from the different kingdoms was analyzed by manual alignment. Examination of the crystal structure of yeast NMT (PDB code: 2P6E) and its structural alignment with human NMT1 (PDB code: 3IU1) were performed using Chimera [28]. For analysis of the N-terminal region of hNMT1, the crystal structures were retrieved from RCSB PDB database [29], using Uniprot ID P30419 (corresponding to hNMT1) as the query. The available structures were manually curated to identify any structural information on the N-terminal portion of molecule.

### Plasmid construction

The plasmids encoding truncated hNMT1s lacking 28 N-terminal amino-acids (Δ28-hNMT1s) were created by standard cloning procedures. The coding DNA for Δ28-hNMT1s was PCR amplified using the forward primer 5’- GCCAAAACCATGAATTCTGCTAGCAAGCGAAGCTAC-3′ (containing a site for EcoRI) and the reverse primer 5′-GAGCGGCCGAAGCTTTTATTGTAGCACCAGTCCAAC-3′ (containing a HindIII recognition site), gel-purified, digested with the appropriate restriction enzymes and cloned into the pETDuet-1Δ6His_hNMT_Nef template (pre-digested with same restriction enzymes) to generate pETDuet-1Δ6His_Δ28-hNMT_Nef. To generate the plasmid for recombinant protein purification (i.e Δ28-hNMT1s without any fusion tags), the region of interest was amplified from p27-hNMT1s using primers 5′‐CTGCCAAACATATGGAGGAGGCTAGCAAG-3′ (forward) and 5′-CAAGCTTGTCGACTTATTGTAGCACCAGTC -3′ (reverse) containing restriction sites for NdeI and SalI, respectively. The protein encoding sequence was cloned into the Nde I and Sal I digested pET27b expression vector (Novagen) generating the recombinant plasmid p27-Δ28-hNMT1s. The recombinant plasmids were verified by DNA sequencing to eliminate the possible introduction of any unintended mutations. A list of all the plasmid constructs used in this study is summarized in S1 Table.

### Metabolic labeling and myristoylation detection by ‘click-chemistry’

To evaluate the myristoyltransferase activity in bacterial cells by co-expression of the NMT gene and a substrate protein, we utilized the recently described dual vector system developed by Glück et al [26]. The full-length catalytic domain of hNMT1 in the vector pETDuet-1Δ6His_hNMT_Nef was replaced with Δ28 N-terminal truncation as described in “Plasmid construction”. The co-expression of genes was followed as described by Glück et al with minor modifications. For expression of the proteins in *E*. *coli* we employed Rosetta 2(DE3) strain. The *E*. *coli* cells transformed with plasmids pETDuet-1Δ6His_Nef, pETDuet-1Δ6His_hNMT_Nef or pETDuet-1Δ6His_Δ28-hNMT_Nef were always maintained with double antibiotic selection, ampicillin (100 μg/ml) and chloramphenicol (40 μg/ml). For protein expression, a single colony was picked from freshly transformed plates and grown overnight at 37°C in LB medium supplemented with appropriate antibiotics as indicated above. The overnight grown culture was used to inoculate fresh LB media (containing the antibiotics) and cultured at 28°C until *A*_600_ reached ~0.6.

For direct comparisons of the protein expression levels, the initial tests of the expression constructs in Rosetta 2(DE3), the cultures were induced with 0.5 mM IPTG. Following induction, the cells were harvested at 6 h (8000 × g, 4°C, 10 min) and the cell pellets were analyzed for protein expression by SDS-PAGE. For metabolic labeling, after the cultures reached an OD_600_ ~0.6 at 28°C, Az-Myr was added directly from a 50 mM stock solution (final concentration 50 μM) followed by additional incubation for 20 min at 25°C. Protein expression was induced at 25°C with 0.5 mM IPTG and the cells were further incubated for 6 hours. Protein expressions were performed with all the indicated plasmids constructs (i.e. pETDuet-1Δ6His_Nef, pETDuet1Δ6His_hNMT_Nef and pETDuet-1Δ6His_Δ28-hNMT_Nef) with or without the addition of Az-Myr. The harvested cells were fluidized in lysis buffer (8.0 mM sodium phosphate, 1.5 mM potassium phosphate, 138 mM NaCl, 2.5 mM KCl adjusted to pH 7.2 and containing 1x EDTA-free complete protease inhibitor cocktail) containing 1% Triton X-100 and lysed by sonication. The lysates were clarified by centrifugation (12000 × g, 4°C, 30 min) and diluted 1:4 with lysis buffer. Nef-His was captured from clarified lysate on Ni-NTA agarose beads (Qiagen) pre-equilibrated with lysis buffer. The beads were washed extensively with lysis buffer and processed for labeling with Alexa Fluor 488 DIBO Alkyne using cyclooctyne azide-alkyne Click-iT Reaction (Invitrogen) according to the manufacturer’s instructions. The click reactions were performed on the proteins bound to Ni-NTA beads. The labeled products were resolved on 12% SDS-PAGE and analyzed by in-gel fluorescence (Molecular Imager FX, Bio-Rad) for verification of the N-myristoylation status of Nef and Coomassie blue stain for the equal protein load.

### Expression and purification of recombinant proteins

For expression of recombinant proteins, *E*. *coli* cells transformed with the plasmid constructs were cultivated at 25°C as described previously [25]. Purification of recombinant Δ28-hNMT1s was performed with minor modifications of the previously described methodology for hNMT1s [25]. In brief, the clarified lysate was applied to a pre-equilibrated SP-sepharose column (GE Life Sciences) with buffer A (50 mM sodium phosphate (pH 7.0) containing 1 mM EGTA and 1 mM EDTA). The column with bound proteins was washed with buffer A containing 80 mM NaCl (6–8 column volumes) to remove the nonspecifically adsorbed material. The protein peak fraction containing Δ28-hNMT1s eluted with buffer A containing 350 mM NaCl. The second step of the purification was performed on size exclusion column chromatography using the Superdex 75 16/60 column (GE Life Sciences) equilibrated with the 50 mM sodium phosphate buffer (pH 7.2) containing 150 mM NaCl, 1 mM EGTA and 1 mM EDTA. This step was carried out with the column coupled to ÄKTA pure device (GE Life Sciences). An isocratic flow rate of 0.5 mL/min was maintained during the chromatographic procedure and the protein elution profiles were monitored by absorbance at 280 nm. SDS-PAGE analysis followed by Coomassie blue protein staining was used for the initial assessment of the purity for enzyme preparations. The concentrations of the purified enzymes were determined spectroscopically using the predicted molar extinction coefficient ε_280_ of 69,330 M^-1^cm^-1^ [25]. All subsequent biochemical assays were performed on the purified recombinant proteins.

### *N-*myristoyltransferase activity assay

The NMT activity was monitored in a coupled enzyme assay monitoring the stoichiometric release of CoA-SH upon formation of the myristoylated peptide [25, 30]. A progressive increase in fluorescence (excitation 384 nm, emission 470 nm) at 15-s intervals over time at 25°C was monitored in solid bottom 96-well black microplate (FLUOTRAC 200, Greiner) using SpectraMax M5 Microplate Reader (Molecular Devices). For relative activity measurements, unless otherwise stated, the standard mixture consisted of 5 nM purified enzyme, 5 μM of MYA and 15 μM CPM. The reaction was initiated by addition of 20 μL of a 100 μM peptide substrate solution (final concentration of 10 μM). All reaction mixtures were 200 μl (final volume) and contained 20 mM sodium phosphate (pH 7.5) with 0.5 mM EDTA, 0.5 mM EGTA and 0.1% (v/v) Triton X-100. Reactions performed in the absence of MYA or peptide served as controls.

For Michaelis-Menten analysis, the reaction velocity (*v*) was obtained at ten different concentrations of substrate catalyzed by the purified samples of hNMT1s and Δ28-hNMT1s at 25°C. The *K*_*m*_ for MYA was evaluated by varying the concentration between 1–10 μM, at a fixed concentration of the assay peptide (50 μM). The *K*_*m*_ with respect to peptide was evaluated by varying its concentration over the range 1–50 μM at a fixed MYA concentration of 30 μM. The background signal in the absence of the respective substrate (over an assay period of 30 min) was subtracted from the signals observed and the substrate-activity data were analyzed to obtain the Michaelis–Menten parameters. Assays were typically performed in triplicate and the mean values obtained were directly fitted to the Eq 1,

$$v=\frac{Vmax\;\times \;[S]}{\text{K}m+[S]}$$ (Eq. 1)

The *K*_*m*_ and *V*_*max*_ values were obtained by nonlinear regression analysis with Origin software (MicroCal).

### Thermal stability analysis

Differential scanning calorimetry (DSC) experiments were carried out on VP-DSC instrument (MicroCal). The purified protein was obtained at a concentration of 0.4 mg/ml in the 50 mM sodium phosphate buffer (pH 7.2) containing 150 mM NaCl. The thermal scans were carried out at 1°C/min from 10°C to 90°C. Thermodenaturation of the investigated proteins was incomplete due to aggregation. The DSC experiments were run at the Biomolecular Interactions and Conformations Facility located at the University of Western Ontario (London, Canada). To further measure the stability profiles of the NMT proteins used in this study, thermal unfolding was carried out by Differential Scanning Fluorimetry (DSF) [31]. The experiments were performed in a final sample volume of 30 μl in 96-well real-time PCR plates. The thermal transition was recorded on a StepOnePlus real-time PCR system (Applied Biosystems). The samples contained 3.3 μM of protein and freshly diluted 5x SYPRO orange dye (Invitrogen) either in the presence or absence of MYA (5 μM) in 50 mM sodium phopspahte buffer (pH 7.0). The fluorescence signals were monitored using ROX settings over a temperature range of 20°C to 90°C with heating rate of 1°C/min. The thermal unfolding curves were analyzed according the two-state transition model given by the relationship:

N→D

where, N and D represents the native and denatured states respectively.

The raw fluorescence signals obtained upon thermal unfolding were baseline subtracted and transformed to obtain the fractional unfolding (F_U_) defined by the Eq 2,

$${\text{F}}_{\text{U}}\;=\frac{f\left(T\right)-f\left(N\right)}{f\left(D\right)-f\left(N\right)}$$ (Eq. 2)

where *f*(*T*), *f*(*N*), and *f*(*D*) are the fluorescence signals, at temperature T, for folded protein and denatured protein, respectively.

The *T*_*m*_ values were determined by curve fitting of the fraction unfolded as a function of temperature directly to the Boltzmann equation (Eq 3),

$${\text{F}}_{\text{U}}\;=\frac{1}{1\;+e^{\left(Tm-T\right)/a}}$$ (Eq. 3)

where *T*_*m*_ is the transition mid-point of thermal unfolding, *T* represents the temperature and *a* denotes the slope of curve within the transition range [31].

### Other analytical measurements

MALDI-TOF, Electrospray ionization mass spectrometry (ESI-MS) and fluorescence spectroscopy (intrinsic tryptophan fluorescence) analysis of the purified Δ28-hNMT1s was performed essentially as described previously for the hNMT1s [25].

## Results and Discussion

### Sequence and structural analysis of the N-terminal region of NMT

**Fig 1:**
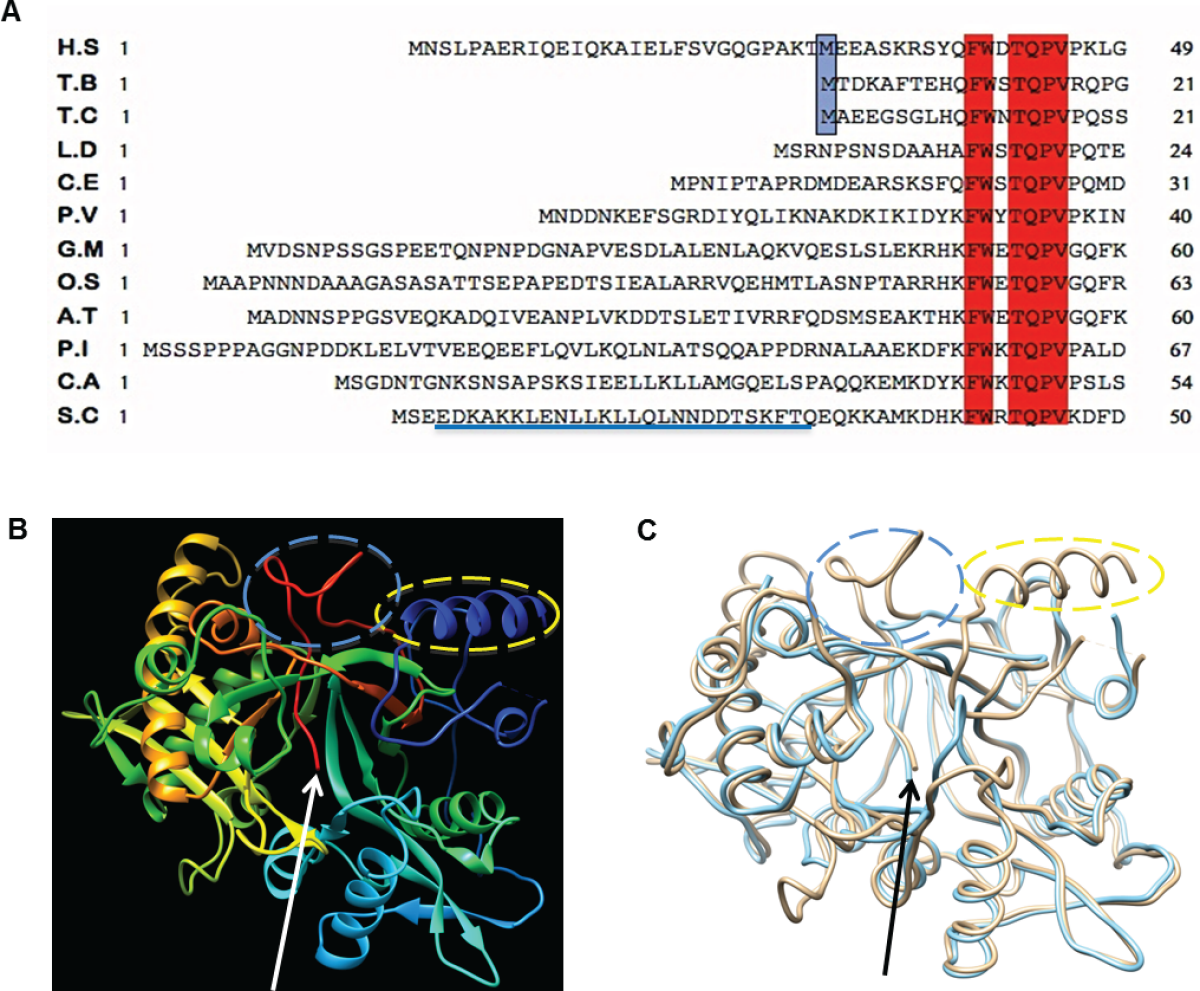
Sequence comparisons of the N-terminal region and structure analysis of NMT. *A*, N-terminal amino acid sequence alignment of orthologous NMT from different species. The comparative alignment of H.S (*Homo sapiens*) sequence is shown with representative organisms T.B (*Trypanosoma brucei*), T.C (*Trypanosoma cruzi*), L.D (*Leishmania donovani*), C.E (*Caenorhabditis elegans*), P.V (*Plasmodium vivax*) A5K1A2), G.M (*Glycine max*), O.S (*Oryza sativa*), A.T (*Arabidopsis thaliana*), P.I (*Phytophthora infestans*), C.E (*Candida albicans*) and S.C (*Saccharomyces cerevisiae*). All sequences were obtained from the SwissProt protein database [27] and their accession numbers are listed under “Materials and methods”. The first conserved residues across all sequences are shown in *red* boxes. The initiator methionine corresponding to Δ28 N-terminal truncation of hNMT1s and its conservation with T.B and T.C NMT is boxed in *light blue*. The sequence region corresponding to the ordered N-terminal region of Yeast NMT is underlined blue. *B*, Ribbon diagram representing the locations of ordered N-terminal region and interacting C-terminal portion in three-dimensional structure model of yeast NMT (Protein Data Bank entry 2P6E, chain C). The ordered α-helical and loop region of N-terminus portion are shown in *blue* and indicated by a dotted ellipsoid (*yellow*). The location of the insertion in C-terminal portion located between βn′ and βo′ (*red*) is indicated by the dotted ellipsoid (*blue*) which is in proximity to the ordered region of N-terminus (shown in *blue* and encircled by *yellow* dotted ellipsoid) *C*, Structural superimposition of the three-dimensional model based structure of yeast and hNMT1s (Protein Data Bank entry 2P6E, chain C and 3IU1, chain A respectively). The *light brown* and *light blue* ribbon traces correspond to yeast and human NMT, respectively. The ordered N-terminal region of yeast NMT is indicated by a dotted ellipsoid (*yellow*) and the differences in the sequence length between βn′ and βo′ regions are indicated by a *blue* dotted ellipsoid. The arrowhead in *B* and *C* indicate the C-terminal tip of the molecule.

The sequence comparisons of the N-terminal region of the catalytic module of hNMT1 with orthologous NMTs reveal that this region varies greatly in length and nature of amino acids (Fig 1*A*). Numerous structural studies of hNMT1 have been performed; however the N-terminal region is not visualized in the reported crystal structures so far. A summary of the available structures and their features are listed in Table 1. A lack of structural information to date in this region of hNMT1 precludes predicting the roles N-terminus plays in terms of interaction with other parts of the molecule or in defining any roles of this region in catalysis. As shown in Fig 1*A*, the [FWXTQPV] motif (3_10_αA’) in the divergent N-terminus represents the first consensually conserved core signature among all NMTs (Fig 1*A*; boxed in red). The 3_10_αA’ motif lies 38 amino acids downstream of the initiator methionine in hNMT1s. This conserved motif also mark the consensually ordered residues observed in most of the crystal structures of hNMT1 listed in Table 1.

**Table 1:** List of hNMT1 crystal structures and their features.

PDB^a^ Code	Resolution (Å)	Bound Ligand	Missing N-terminus^b^
**1RXT**	3.0	None	1–81
**3IU1**	1.42	MYA	1–34
**3IU2**	1.73	MYA; Inhibitor	1–34
**3IWE**	1.79	MYA; Inhibitor	1–34
**3JTK**	1.61	MYA; Inhibitor	1–34
**4C2Y**	1.64	MYA	1–34
**4C2Z**	2.08	MYA; Inhibitor	1–34

^a^ PDB, Protein Data Bank.

^b^ The residue numbering corresponds to the equivalent residues in the catalytic module of hNMT1 (i.e hNMT1s).

The shortest N-terminus sequence length upstream of 3_10_αA’ is presented by *Trypanosoma brucei* and *Trypanosoma cruzi* (Uniprot ID Q388H8 and Q4E2V2, respectively) (Fig 1*A*). These kinetoplastid parasites, which are the lower organisms in eukaryotic evolution, illuminate the development of more complex regulatory systems in higher eukaryotes. Given the dynamic nature of residues and varying degree of sequence length, it appears that the N-terminal part of the protein is not crucial for catalysis but is involved in regulating NMT functions. The lower eukaryotes, *T*. *brucei* and *T*. *cruzi*, lack the N-terminal region corresponding to the first 28 residues of hNMT1s. The NMTs from these organisms provide with good models to compare and study evolution of functional complexity in the corresponding enzyme from higher organisms. The sequence comparisons reflect that an internal methionine is available at an analogous position in the hNMT1s (Fig 1*A*; boxed in light blue). This suggests that a shorter domain organization may define a completely functional module in the human enzyme.

The *S*. *cerevisie* enzyme is one of the best-characterized members of NMT family and the recently solved crystal structure of yeast NMT (PDB code: 2P6E) reflect that residues from N-terminal segment of the protein folds as α-helix (αB′) and a loop (B′A′) (Fig 1*B*). This portion plays an important role in the binding of both MYA and peptide substrate and also helps to stabilize the overall structure of the enzyme [32]. The structural analysis of *S*. *cerevisie* reveals that the ordered N-terminal portion of the yeast NMT is in close contact with the C-terminal half of the molecule involved in peptide binding (Fig 1*B*). The interaction with distal C-terminal involves the region corresponding to a prominent insertion located between βn′ and βo′ in the C-terminal portion of the molecule [33]. This portion of the molecule is protruded from the main body of the protein (Fig 1*C*). However, at the structural level, the interaction interface of the yeast NMT is fundamentally different from the orthologous human enzyme. In humans, a much shorter stretch of amino acids connects the strand βn′ to the structurally equivalent βo′ in C-terminal portion [33]. Accordingly, the protrusion observed in the yeast counterpart is absent from the human enzyme (Fig 1*C*). Thus it is plausible, that the dynamics of interaction of the N-terminus in human enzyme is entirely different from that of the yeast NMT. Here, by tuning the sequence deletion to the minimal catalytic boundary at comparable sequence length at N-terminus defined by *T*. *brucei* and *T*. *cruzi* NMT and in combination with biochemical and biophysical analyses, we demonstrate *in vitro* what regulation this atypical extension confers on to NMT activity.

### The amino–terminus of hNMT1s is dispensable for enzymatic activity

NMTs belongs to GCN5-related N-acetyltransferase superfamily of proteins and operates though an ordered bi-bi reaction mechanism [34]. The NMTs from diverse organisms adopt the same catalytic mechanism and thus have an architecturally conserved core in consistence with the precise identity and positioning of residues required for binding and catalysis [32–34]. The binding of MYA is an essential prelude to the binding of the substrate peptide following which the catalysis occurs by a nucleophilic addition-elimination reaction [2, 5]. The C-terminal main chain carboxylate functions as a catalytic base to deprotonate the amino-terminus glycine (Gly1) ammonium of peptide substrate. The deprotonated Gly1 forms the nucleophilic amine to attack the polarized thioester carbonyl of MYA subsequently leading to the formation of covalently linked myristoylated peptide [2, 5, 34, 35]. Previous observations in the N-terminus of hNMT1s (i.e the defined catalytic module of 416 amino-acids) have indicated that addition of N-terminal extensions serve to stabilize the MYA:NMT intermediate complex [36]. However, in consistence with essential role of C-terminal carboxylate in catalysis, NMTs are intolerant to deletions at the C-terminus portion of the molecule [35]. Given the divergence in sequence length observed at the N-terminus portion of the orthologous NMTs (Fig 1*A*), we reasoned that the sequence length at N-terminal of the NMT enzyme is not crucial for catalysis and thus hNMT1s should be amenable to fine tuning for defining the minimal catalytic core. Taking information from the lower eukaryotic counterpart, we have focused on the N-terminal boundary defined by the equivalent positions in *Trypanosoma* NMT.

To explore the role of amino-terminus on NMT enzymatic activity we first established, in an *E*. *coli* complementation assay, that the Δ28-hNMT1s is catalytically active *N*-myristoyltransferase. *E*. *coli* does not harbor a system for N-myristoylation (either endogenous NMT or suitable substrates) but is able to myristoylate substrate proteins when co-expressed with functional N- myristoyltransferase [37, 38]. The myristoylation of substrate proteins in the presence of exogenously added myristic acid provides an excellent system for identification of functional myristoyltransferase or myristoylated proteins in a cellular milieu [39].

**Fig 2:**
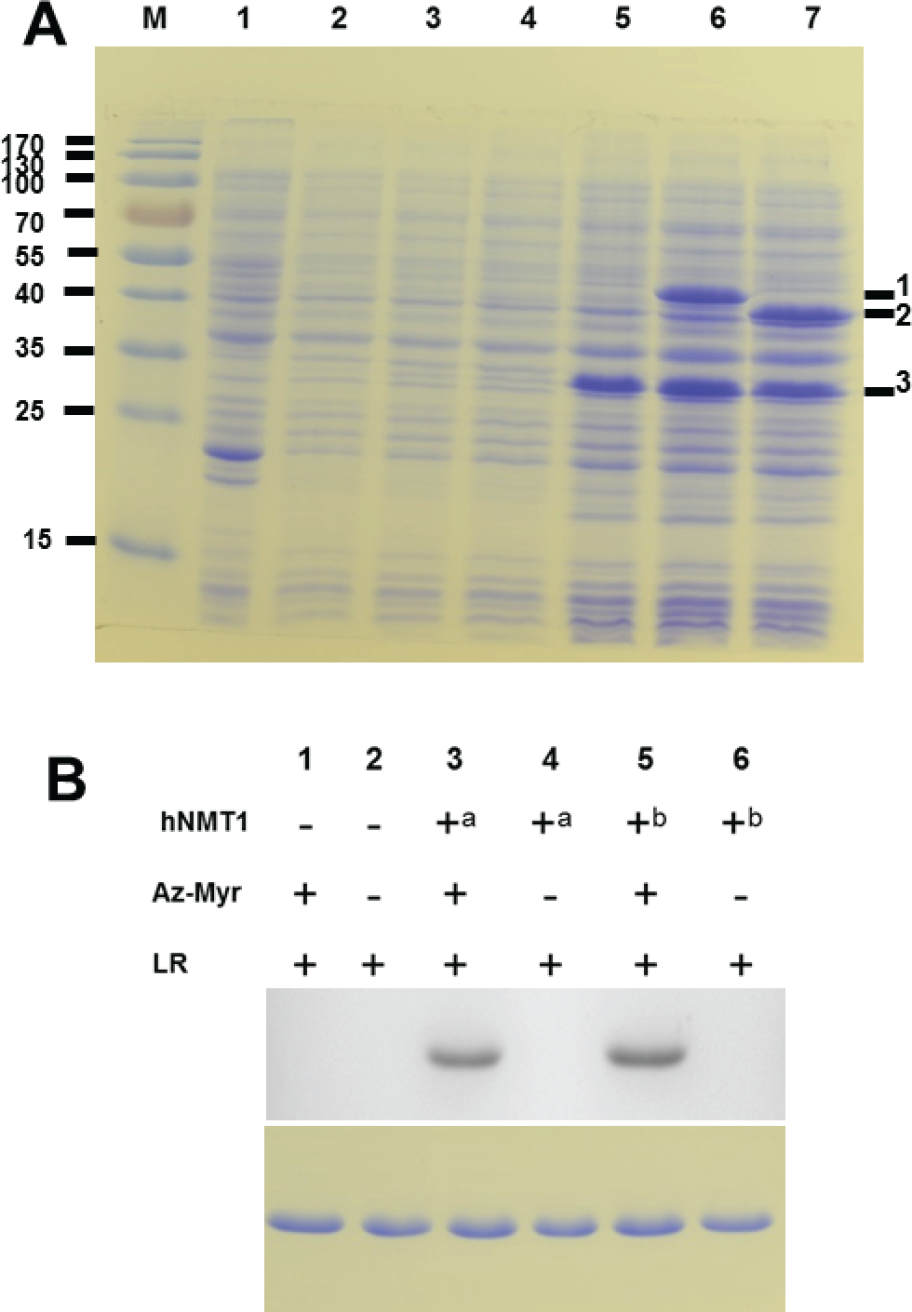
Analysis of *N*-myristoyltransferase activity in *E*. *coli* cells by complementation assay. *A*, SDS-PAGE analysis of Nef and NMT expression. The plasmid constructs encoding either Nef alone or with NMT transformed in Rosetta 2(DE3) cells were analyzed for expression, 8 hours after IPTG induction. Lane “M” corresponds to protein molecular weight marker, lane “1” control lysate from Rosetta 2(DE3) cells, lanes “2–4” and “5–7” pre and post-induction samples transformed with plasmids pETDuet-1Δ6His_Nef, pETDuet-1Δ6His_hNMT_Nef and pETDuet-1Δ6His_Δ28-hNMT_Nef respectively. The proteins hNMT1s, Δ28-hNMT1s and Nef are marked ^1, 2^ and ^3^. *B*, Detection of *N*-myristoyltransferase activity in *the E*. *coli* complementation assay by metabolic labeling with bio-orthogonal myristic acid analogue. Myristoylation of Nef was evaluated by labeling with Az–Myr followed by ‘click-chemistry’ detection with Alexa Fluor 488-labelled DIBO alkyne as described in “Materials and methods”. The in-gel fluorescence signals (top panel) from myristoylated Nef-His isolated from *E*. *coli* cells either not expressing NMT (lane 1 and 2), expressing hNMT1s (lane 3 and 4) or expressing Δ28-hNMT1s, (lane 5 and 6) treated with labeling reagent (LR) (tagged with or without Az–Myr) as indicated. ^a^ and ^b^ correspond to hNMT1s and Δ28-hNMT1s respectively. The input levels of Nef-His were determined by Coomassie blue staining of the SDS–PAGE gel run in parallel (lower panel).

Towards this goal, we utilized the recently described single vector system that utilizes a bicistronic vector for the co-expression of human *N*-myristoyltransferase 1 (*hNMT-1*) gene and HIV-1 negative regulatory factor (*nef)*, a substrate protein for *hNMT-1* [26]. To facilitate purification, the constructs encode Nef protein as a fusion to 6x-His tag at the C-terminus of the molecule. We replaced the full-length catalytic domain (i.e hNMT1s) in pETDuet-1Δ6His_hNMT_Nef with Δ28-hNMT1s (renamed as pETDuet-1Δ6His_ Δ28-hNMT_Nef) and subsequently verified for the expression of the NMT and Nef. In parallel, the constructs encoding the *nef* gene with and without the complete catalytic module of hNMT1 (pETDuet-1Δ6His_hNMT_Nef and pETDuet-1Δ6His_Nef, respectively) were also evaluated simultaneously. After induction with IPTG, comparable levels of Nef expression was achieved in all the constructs transformed in Rosetta 2(DE3) cells as demonstrated by SDS-PAGE (Fig 2*A*, lanes 5–7). The molecular weight of expressed Nef is about 24.6 KDa but the protein shows an anomalous migration which corresponded to a higher molecular mass of ~30 KDa (Fig 2*A*, lanes 5–7). However, this is in consistence with the noted observations of the migration behavior of Nef expressed in *E*. *coli* cells [26, 40]. As shown in Fig 2*A*, an additional band arises after induction of the cells transformed with pETDuet-1Δ6His_hNMT_Nef and pETDuet-1Δ6His_Δ28-hNMT_Nef, but not with pETDuet-1Δ6His_Nef, indicating co-expression of the NMT. The additional band in pETDuet-1Δ6His_hNMT_Nef construct runs at a position corresponding to the molecular weight of ~ 48 kDa corresponding to the molecular weight of the full-length catalytic domain of *h*NMT1 (Fig 2*A*, lane 6). However, in the constructs encoding pETDuet-1Δ6His_Δ28-hNMT_Nef, the additional expression band has a clearly distinguishable faster migration behavior (corresponding to the truncation of ~ 3 kDa) on the SDS-PAGE, indicating successful co-expression of the Δ28-hNMT1s (Fig 2*A*, lane 7).

We further coupled the single vector expression system with the ‘click-chemistry’ labeling for identification of myristoylated Nef [38]. The ‘click-chemistry’ involves the metabolic labeling of cells with azido or alkynyl fatty acid analogues followed by reaction of modified proteins with chemoselective detection tags. The azide conjugated myristic acid analogue (i.e Az-Myr) was added to cells ~20 min before IPTG induction to a final concentration of 20 μM. The C-terminal His-Nef was expressed alone or in conjugation with the *h*NMT1 gene (constructs described above) both in the presence and absence of the exogenously added Az-Myr. The expressed Nef-His was captured from the clarified bacterial lysate on Ni-NTA beads and allowed to react with strain-promoted labeling reagent Alexa Fluor 488 DIBO Alkyne. The myristoylation status of expressed Nef upon induction was validated by visualization of the fluorescent signal by an in-gel fluorescence assay. The substrate Nef was labeled only when the NMT was present and Az-Myr was added to the culture medium (Fig 2*B*, lane 3 and 5; top panel). The equivalent expression levels of Nef were determined by Coomassie blue stain (Fig 2*B*; lower panel). The proof of successful myristoylation of Nef by Δ28-hNMT1s was demonstrated by the chemoselective labeling of Nef with labeling reagent in the presence of exogenously added Az-Myr (Fig 2*B*, lane 3 and 5; top panel). This validates that the N-terminal truncation of hNMT1s does not affect its catalytic ability to transfer the myristoyl moiety from MYA to the deprotonated Gly1 ammonium of the substrate molecule.

### Expression, purification and characterization of the recombinant proteins

**Fig 3:**
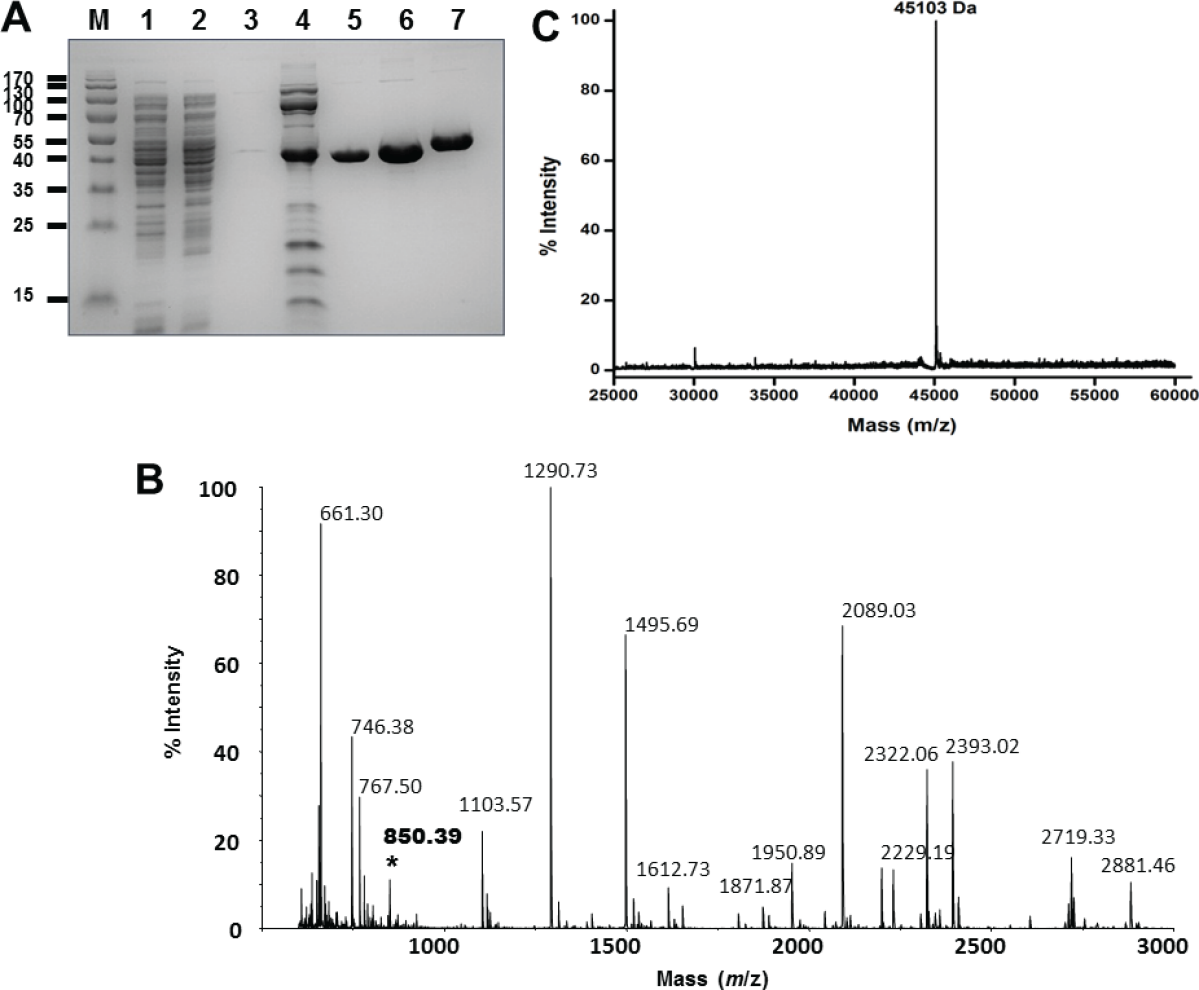
Purification and analytical investigations of the proteins used in study. *A*, Coomassie blue stained 12% SDS-PAGE analysis of NMT proteins. Lane “M” represents protein bands of the applied molecular weight marker and is labeled with the corresponding molecular weight in kDa. A typical expression profile of Δ28-hNMT1s is shown in lanes 1–6 wherein lanes 1 and 2: aliquots of total cell lysate from expression in two representative batches; lanes 3: protein content in wash fraction; lane 3: protein eluted from ion-exchange chromatography; lanes 5 and 6: 5 and 15 μg protein aliquot after the final purification step on Superdex 75 gel-filtration column. For purity assessments, lane 7 shows 10 μg protein aliquot of hNMT1s purified in parallel. The purifications yielded proteins with purity >95%. *B*, MALDI-TOF mass spectrometry analysis of Δ28-hNMT1s. The peaks corresponding to deletion at the N-terminal region of hNMT1s were missing and are described in text. The peak of 850.39 Da (marked in boldface with *) results from the peptide [MEEASKR] at the engineered N-terminus. *C*, ESI-MS analysis of the Δ28-hNMT1s with no indications for presence of any degradation or aggregation product. The measured protein mass of 45103 Da exactly matches the calculated mass.

To assess the effect of the N-terminal amino acids on hNMT1s activity and for further biochemical investigations, we next intended to produce the truncation mutant devoid of any fusion tags. Towards this end we engineered and cloned the Δ28-hNMT1s in the T7-based expression vector pET27b (Novagen). The residue at position 29 was chosen as the consensus start site as it was in equivalent positions to the initiator N-terminal methionine of the NMT polypeptide of *T*. *brucei* and *T*. *cruzei* which had the minimal sequence extension ahead of the first ordered residues [FWXTQPV] in the consensually conserved 3_10_αA’ motif (Fig 1*A*). After expression in *E*. *coli* BL21 (DE3) cells, Δ28-hNMT1s showed a faintly detectable band on the SDS-PAGE (Fig 3*A*, lane 1 and 2). However, employing the simplified purification protocol as described previously [25], we recovered >95% pure homogenous protein after a two-step chromatographic purification on an ion-exchange resin followed by gel-filtration chromatography (Fig 3*A*, lane 5 and 6). The final yield of the purified recombinant Δ28-hNMT1s enzyme was ~8 mg Litre^-1^ of the expression culture as obtained earlier for the hNMT1s enzyme [25]. For comparative assessment of the protein properties, hNMT1s was expressed and purified in parallel (Fig 3*A*, lane 7). The Coomassie stained purified proteins are displayed on 12% SDS- PAGE in Fig 3*A*. The truncation mutant Δ28-hNMT1s shows a distinctly observable faster migration rate than the full-length hNMT1s in correspondence with the shorter sequence length at N-terminus (Fig 3*A*, lane 5 and 6 vs. 7). An overlay of the gel filtration elution profiles of Δ28-hNMT1s with hNMT1s demonstrated that both proteins resolve at identical elution volumes suggesting a similar globular size and thus indicating that there were no changes in the oligomerization status of the protein due to the truncation of N-terminal region (S1 Fig).

Peptide mass fingerprint analysis utilizing MALDI-TOF was performed on the in-gel tryptic digest of the Δ28-hNMT1s and the mass spectra is presented in Fig 3*B*. The spectra reflected that the peaks corresponding to the masses 917.45 and 1316.72 observed in the mass spectra of hNMT1s are not noticeable [25]. The 917.45 and 1316.72 Da corresponds [M+H]^+^ ions of the tryptic fragments corresponding to the peptides [MNSLPAER] and [AIELFSVGQGPAK] in the N-terminal region and are sharply observed in the mass spectrum of the full-length protein [25]. The MALDI-TOF spectrum of the Δ28-hNMT1s shows an additionally unique peak of 850.39 Da that results from the [M+H]^+^ ion of the peptide [MEEASKR] corresponding to the tryptic fragmentation of the engineered N-terminus and was earlier not reflected in the mass spectrum of the full-length protein [25]. These observations validate the authenticity of N-terminal truncation of hNMT1s. The intact mass analysis of the purified Δ28-hNMT1s protein by ESI-MS reflected a single prominent component at the mass of 45103 Da (Fig 3*C*). This most accurately validates the calculated theoretical mass of 45102.04 Da and indicates that the protein was purified in a highly pure form as that of the full-length catalytic domain utilizing our approach described earlier [25].

### The N-terminal deletion enhances myristoyltransferase activity of hNMT1s

The NMTs exhibits pseudo two-fold symmetry with two distinct domains in which the N-terminal half forms the MYA binding pocket and the C-terminal half forms the major portion of the peptide substrate binding [2, 5, 32–34]. The binding of MYA to the N-terminal half is an essential penultimate step to allow for the binding of peptide substrate and thereafter transfer of myristate moiety to the NH_2_ group of Gly1 on peptide substrate [5]. Since the N-terminal half of NMT is involved in MYA binding, to explore the role of the truncation in the N-terminal region in NMT function, we first evaluated whether the deletion of N-terminal stretch of hNMT1s alters the MYA binding or recognition. To investigate, we employed an assay to monitor the covalent complex formation of NMT:MYA that exploits the quenching of intrinsic tryptophan fluorescence of NMT upon MYA ligand binding [25]. The deletion mutant was incubated with molar excess of MYA and fluorescence measurements were carried out as described earlier for hNMT1s [25]. The quenching of fluorescent signals for the Δ28-hNMT1s (S2 Fig) were identical to that observed earlier for the hNMT1s [25]. The observation indicated that the deletion of the N-terminal region of hNMT1s does not alter its myristoyl-CoA complex formation step, a key prerequisite for catalysis to occur.

**Fig 4:**
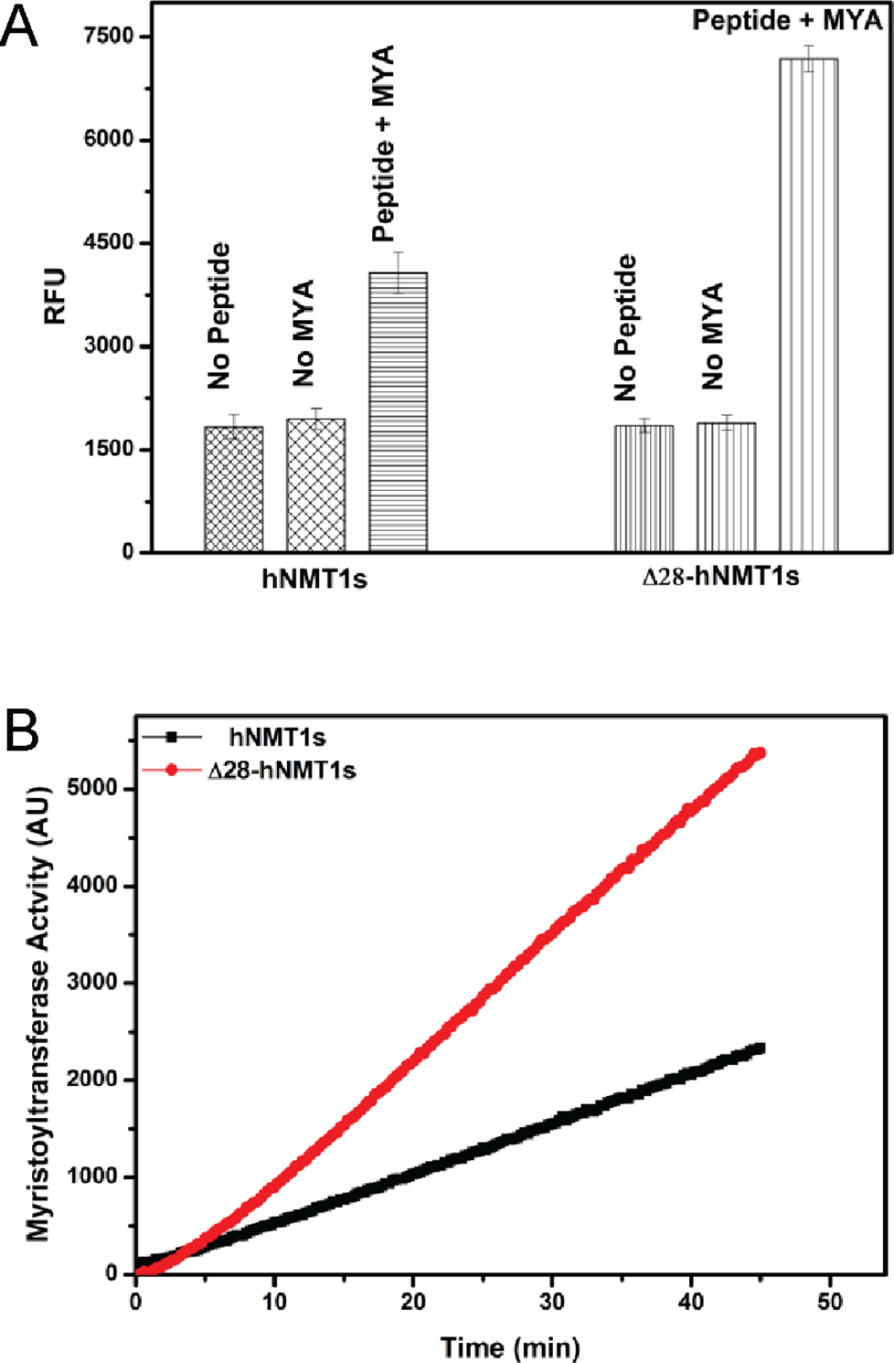
Comparison of the effects of N-terminal deletion on myristoyltransferase activity. *A*, Biochemical analysis of the enzymatic property of hNMT1s and Δ28-hNMT1s. Purified enzymes (5 nM) were assayed in standard reaction as described under “Materials and methods”. The resultant increase in fluorescence (λ_ex_ 384 nm, λ_em_ 470 nm) was quantified in presence or absence of either of the substrates in an assay time of 30 min at 25°C. Enzyme activity is expressed as relative fluorescence units (RFU) generated in the reaction. Values represented are mean ± S.D of triplicate measurements. *B*, Enhancement of the myristoyltransferase activity by N-Terminal deletion. Myristoyltransferase activity was monitored by the purified enzymes (5 nM) at saturating concentration of both substrates (30 μM each) in 200 μl of buffer containing 20 mM sodium phosphate (pH 7.5), 0.5 mM EDTA, 0.5 mM EGTA and 0.1% (v/v) Triton X-100. The fluorescence signals (λ_ex_ 384 nm, λ_em_ 470 nm) generated were recorded progressively at 15-s interval and are reported over the linear range of responsiveness. The represented kinetic traces are the average of three measurements after subtraction of blank signals (reactions performed in the absence of the acceptor peptide substrate).

To further substantiate, how the deletion of N-terminal domain of hNMT1s affects its potency to mediate the transfer of myristoyl group from the donor MYA ligand to acceptor peptide substrate, an *in vitro* enzymatic assay was employed to monitor the NMT activity. We observed that the myristoyltransferase activity of the Δ28-hNMT1s was enhanced in comparison to the hNMT1. In control experiments, lacking either MYA or peptide, no enhancement was detected (Fig 4*A*). The signals in the absence of the co-substrates (either MYA or peptide) reflect the background fluorescence from the reactivity of CPM in the assay reagent to the protein sulfhydryl groups. The identical signal intensity values generated both in the hNMT1s and Δ28-hNMT1s (in the absence of either MYA or peptide) confirms similar protein concentrations used in the assay and precludes any erroneous addition of higher enzyme amounts.

To account that the observed effect was not a consequence of the limiting substrate concentrations, the enzymatic assays were performed at several fold molar excess of both co-substrates (30 μM each) and a linear enhancement effect was observed (Fig 4*B*). The observed findings indicate that the deletion of N-terminus benefits the ability of hNMT1s to transfer the myristoyl moiety to the acceptor peptide substrate. Under identical reaction conditions (reaction time 45 min), ~2.3 fold in increase in the myristoyltransferase activity was observed (Fig 4*B*).

### The N-terminal Δ28-deletion mutation does not alter the stability of the enzyme

**Fig 5:**
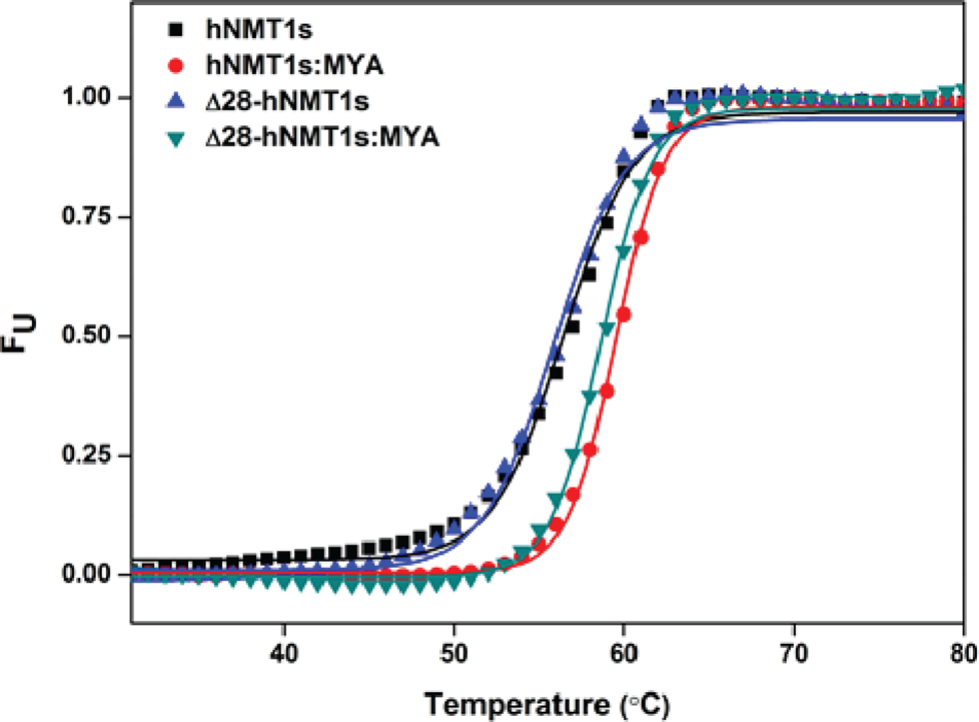
Thermal unfolding of variants hNMT1s and Δ28-hNMT1s. The protein unfolding was monitored by the binding of Sypro orange dye to exposed hydrophobic patches upon thermal transition. The fluorescence signals were recorded at 3.3 μM of protein and 5x SYPRO orange dye (either in presence or absence of 5 μM MYA) in 50 mM sodium phosphate buffer (pH 7.0). The symbols represent the values obtained at each temperature and the fitted curves represent the Boltzmann fitting of the data as described under “Materials and methods”.

The organization of the N-terminal region is poorly understood, presumably due to its inherent flexibility and thus lack of any structural information at the heterogeneous amino terminal region in the reported crystal structures so far (Table 1). It is widely understood that the protein stability increases due to decrease in the entropy of unfolding. One explanation for increased activity of the Δ28-hNMT1s in comparison to hNMT1s is that it may have conferred increased stability due to the loss of the flexible N-terminal portion of the hNMT1s. We therefore aimed to investigate the thermodynamic stability profiles of both hNMT1s and Δ28-hNMT1s. Differential scanning calorimetry is perhaps the most informative technique to analyze the complete behavior of molecules upon thermal unfolding [41]. However, our initial attempts to perform thermal stability studies by DSC showed that the protein precipitates immediately after denaturation (S3 Fig). This makes the denaturation an irreversible process that restricted us to obtain the complete thermodynamic profile by DSC. We therefore next compared the stability of the full-length and truncated protein by measuring the *T*_*m*_ by DSF analysis using the SYPRO orange dye. The process monitors the change in fluorescent signals as the dye interacts with the exposed hydrophobic patches of the protein undergoing thermal transition [31]. We observed that both enzymes (ie. hNMT1s and Δ28-hNMT1s) have identical thermostability as reflected by their very similar *T*_*m*_ values in their *apo* form and an equivalent modest increase in the MYA bound states (Fig *5*). The *T*_*m*_ values obtained from the thermal stability analysis are summarized in Table 2. The observations indicate that the higher activity of the Δ28-hNMT1s over hNMT1s is not due to any enhancement of the protein stability because of the deletion of the N-terminal segment.

**Table 2:** Thermal stability parameters for full-length catalytic domain (hNMT1s) and N-terminal truncated (Δ28-hNMT1s) human *N*-myristoyltransferase 1 in *apo* and MYA bound states.

Enzyme	T_m_ (°C)		Δ Tm (°C)
	*apo*	MYA	MYA- *apo*
hNMT1s	56.43±0.20	59.52±0.07	3.09
Δ28-hNMT1s	55.87±0.22	58.63±0.11	2.76
Difference	0.56	0.89	-

The values depicted are from the curve fitting of the fraction unfolded (F_U_) as a function of temperature directly to the Boltzmann equation, described by Eq 3 under “Materials and methods”.

### Differential response of Δ28-NMT1 to the *N*-myristoyltransferase substrates

**Fig 6:**
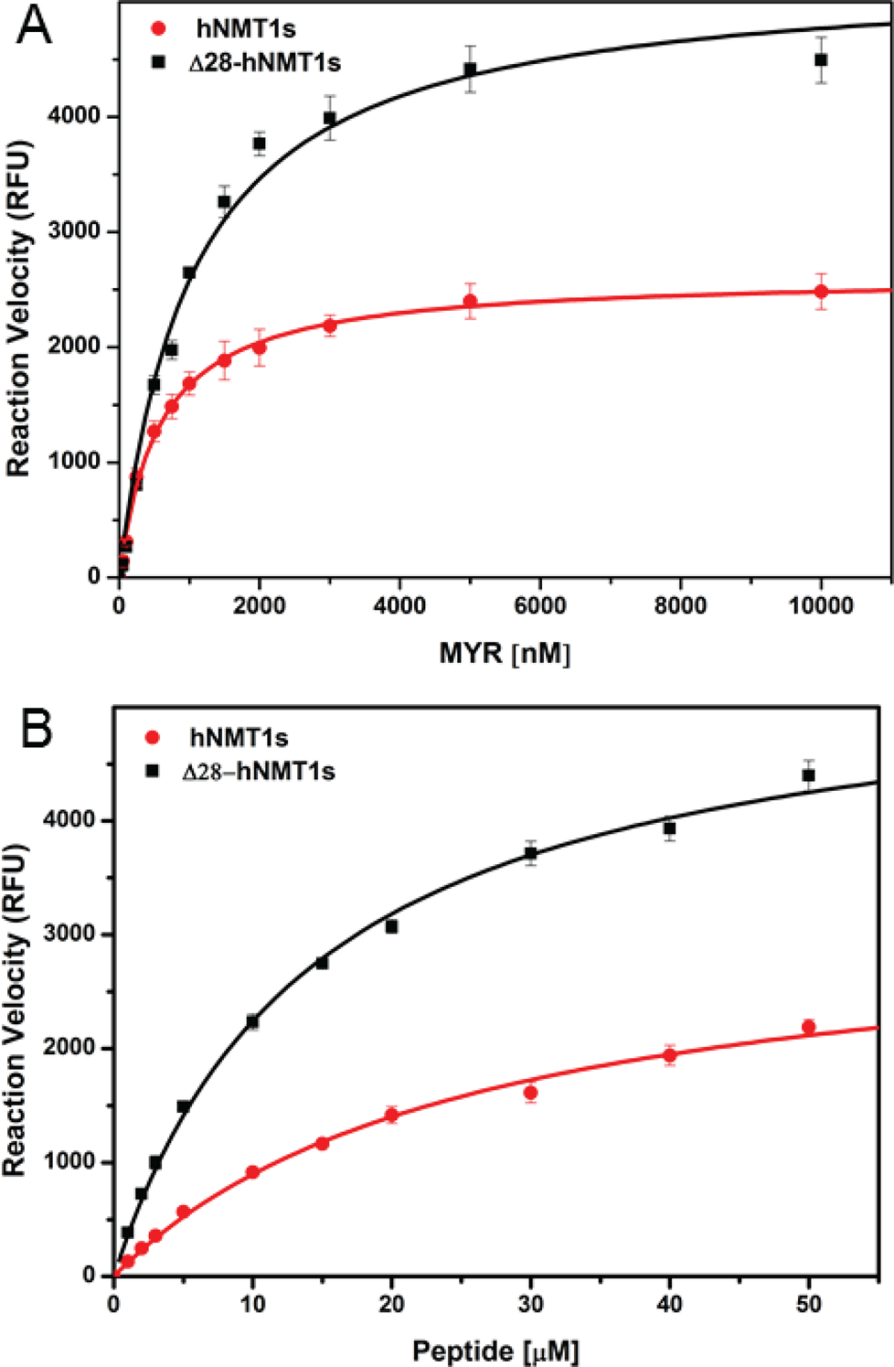
Analyses of catalytic efficiencies of hNMT1s and Δ28-hNMT1s. Enzymatic activity of 5 nM of hNMT1s and Δ28-hNMT1s was assayed in 200 μl of 20 mM sodium phosphate buffer (pH 7.5) with 0.5 mM EDTA, 0.5 mM EGTA, and 0.1% (v/v) Triton X-100 at 25°C using the fluorescence assay as described under “materials and methods”. Plots of reaction velocity versus substrate concentration were subjected to Michaelis-Menten analysis as defined by Equation 1 under “materials and methods”. ***A*,** Michaelis-Menten analysis of MYA as a substrate of NMT catalyzed myristoylation at a peptide substrate concentration of 50 μM and ***B*,** Michaelis-Menten analysis of peptide substrate by NMT catalyzed myristoylation at a MYA substrate concentration of 30 μM. Errors bars represent the standard deviation and are from triplicate measurements. The Michaelis constant (*K*_m_) and its standard deviation provided under Table 3 are from the results of the curve fitting by nonlinear regression analysis to the data points.

To explore the extent to which the truncation of N-terminal region impacts the kinetic features of hNMT1s, the enzymatic properties of hNMT1s and Δ28-hNMT1s were evaluated using the real time kinetic assay described under “Materials and methods”. Since the N-terminal half is reported to be involved in MYA binding [34], we first evaluated the kinetic parameter for MYA at fixed peptide concentration of 50 μM. A clearly distinguishable enhancement in *V*_max_ (~ 2 fold) was observed for MYA upon the N-terminal truncation (Fig 6*A*). However, the enzyme kinetics analysis showed that the Michaelis constant for the MYA was reduced by the Δ28-hNMT1s in comparison to hNMT1s (Table 3). In the crystal structures of yeast NMT the N-terminal portion shows interaction with α-helical protrusion located between strands βn′ and βo′ in the distal C-terminal segment, the region involved in formation of peptide binding site (Fig 1*B*) [32]. We therefore, next investigated whether the N-terminal truncation of hNMT1s has any effects on the kinetic behavior of the peptide substrate. We observe that the truncation of the N-terminal region of hNMT1s enhanced the peptide binding affinity (Table 3); a pocket located in the distal C-terminal portion of the molecule, and also enhanced the *V*_max_ ~ 1.7 fold (Fig 6*B*). However, since the *K*_m_ values also seem to change concurrently with the enhancement in the *V*_max_, we evaluated the change in enzymatic efficiency (i.e *V*_max_/*K*_m_) towards both MYA and peptide to more precisely understand the effects of N-terminal truncation. A comparison of the relative enzymatic efficiency shows that the N-terminal truncation mutant reflects a 1.09 fold and 3.05 fold difference of this parameter for MYA and peptide, respectively. This indicated that the truncation does not alter the enzymatic efficiency towards MYA but increases towards the peptide substrate. A comparative summary of these parameters for hNMT1s and Δ28-hNMT1s is presented in Table 3. Our findings suggest that the deletion of the N-terminal amino acids delimited by the sequence boundaries defined by orthologous NMT from the lower eukaryote *T*. *brucei and T*. *cruzi* are expendable in relation to activity. Overall, given the higher inherent activity of Δ28-hNMT1s, our data indicates that the N-terminal residues of hNMT1s confer autoinhibition by interacting with peptide-binding portion of the protein core in the C-terminal region. Accordingly, the deletion of N-terminus prevents the autoinhibiting interaction, removing the barrier to maximal catalytic activity, thus explaining the elevated activity of Δ28-hNMT1s as compared to hNMT1s.

**Table 3:** Kinetic analysis of the myristoylation reaction by full-length catalytic domain (hNMT1s) and N-terminal truncated (Δ28-hNMT1s) human *N*-myristoyltransferase 1.

Enzyme	MYA		Peptide	
	*K*_m_ (nM)	Relative *V*_max_/*K*_*m*_	*K*_m_ (μM)	Relative *V*_max_/*K*_*m*_
hNMT1s	568.17±32.06	1	25.69 ±2.57	1
Δ28-hNMT1s	1038.32±122.31	1.09	14.39±0.97	3.05

### Physiological significance of N-terminal processing

N-Myristoylation was initially sought to be a co-translational process that occurred on terminal glycine residues while the translating polypeptide remains bound to the ribosome [2, 42]. In consistence with the co-translational myristoylation process, the human NMTs have an extended N-terminal portion containing a poly-lysine cluster which helps it to tether to the ribosome [24]. However, subsequently it has been documented widely that N-myristoylation is not only a co-translational process but also occurs on many proteins in post-translational fashion [3, 43, 44]. This occurs mostly in apoptotic states, following a proteolytic cleavage, which results in an unmasking of an internal glycine residue [3, 43]. Physiologically, cells undergoing apoptosis would have a high demand for myristoylation as a disproportionate number of substrates become available very quickly and require to be N-myristoylated to perform their physiological functions. To meet the increased demands for myristoylation of substrates, cells could undergo an increased synthesis of the NMT enzyme. However, given the rapid time scales of apoptosis, the synthesis rate of the polypeptide may not keep up with the higher requirements.

We suggest that the uncoupling of the N-terminal regulatory regions of NMT thus might serve as a mechanism to overtake the loads of myristoylation demands. Our speculation is corroborated by the fact that hNMT1 is observed to undergo proteolytic processing in its N-terminus region in the apoptotic states [45, 46]. Differential processing resulting in diverse N-terminal regions corresponding to cleavage at amino acid positions 20 and 35 in the catalytic domain has been observed by Mahrus et al [45]. These physiological isoforms generated during the apoptotic states closely serve the domain boundaries of Δ28-hNMT1s employed in our *in vitro* studies. A physiological counterpart for the hNMT1s is reflected by the isoform generated by Caspase 3 mediated proteolytic processing at amino acid position 72 in the ribosome-targeting domain [46]. We suggest that the N-terminal processing of NMT *in vivo*, may directly serve to rapidly meet the high turnover rates for increased myristoylation demands of substrates protein generated by apoptotic processing.

## Conclusions

Evolution shapes the functional landscape of proteins by introduction of mutational variations to achieve species-specific roles. Such mutations are mostly observed within the loops or surface regions to attain substrate specificity and very often do not perturb the structural core of protein. However, as the complexity evolves from lower to higher organisms, variations among the functionally equivalent enzymes are observed by insertions of non-catalytic domains, which serve the regulatory roles and are non-essential for catalysis. Here we have demonstrated that the enzyme hNMT1 in its catalytic domain possess amino acids segments which are redundant for its enzymatic activity. We verified this postulate by engineering a hNMT1s truncation mutant devoid of the N-terminal 28 amino acid and showed *in vitro*, that the deletion significantly enhances myristoyltransferase activity without any compromise in stability of the enzyme. In conjugation with the earlier *in vivo* observations, this enhanced activity may serve the requirements to meet the increased demands for myristoylation in diverse cellular states.

## Supporting Information

S1 Fig Size-exclusion chromatogram of the proteins used in study. Superdex 75 gel filtration elution profiles for the proteins performed in the buffer system 50 mM sodium phosphate (monobasic); pH 7.2, 150 mM NaCl, 1 mM EGTA and 1 mM EDTA. The identical elution profile of hNMT1s and Δ28-hNMT1s in comparison to ovalbumin (Mol. wt. 48.1, 45.1 and 43.0 kDa respectively) indicates that the expressed proteins are monomeric in nature.
(TIF)

S2 Fig Fluorescence quenching of Δ28-hNMT1s upon MYA binding. Ligand binding induced quenching of the intrinsic tryptophan fluorescence of Δ28-hNMT1s was performed in 20 mM HEPES buffer; pH 7.2 and 50 mM NaCl at protein concentration of 1 μM in presence or absence of MYA (as indicated). The spectrum was recorded from 300–450 nm (λ_ex_ 295 nm).
(TIF)

S3 Fig DSC scan for thermal denaturation of hNMT1s. The sharp decline after the inflection point of the curve indicates aggregation of the investigated protein. The black line depicts the heat signals while red line is the single peak fit to the observed unfolding.
(TIF)

S1 Table List of plasmids used in the study and their characteristics. (DOC)
