# Supplementary figures and images for: N-Terminal Region of the Catalytic Domain of Human N-Myristoyltransferase 1 Acts as an Inhibitory Module
Source: PLoS One. 2015 May 22;10(5):e0127661. doi: 10.1371/journal.pone.0127661 (PMC4441422; doi:10.1371/journal.pone.0127661)

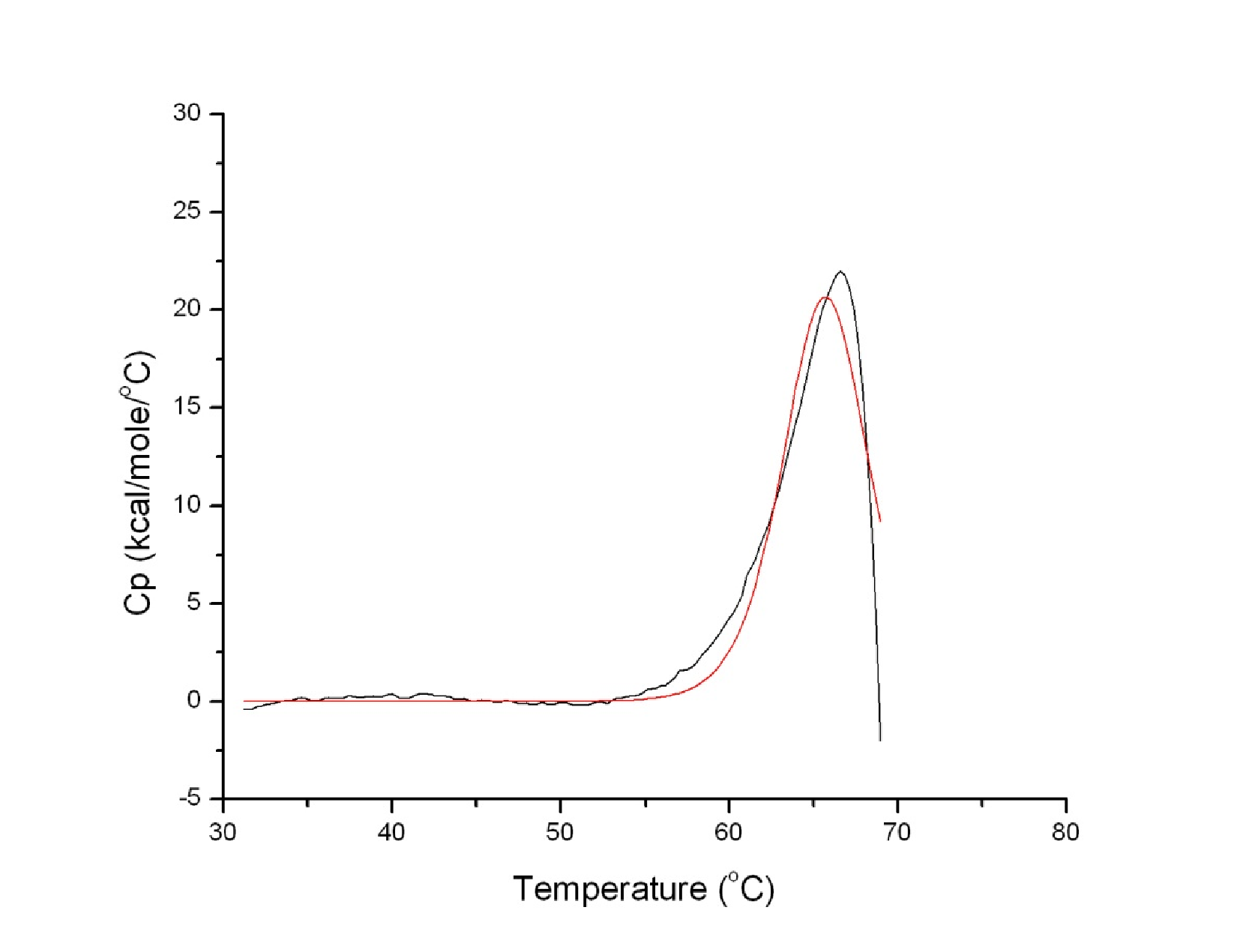

Supplement: S3 Fig — The sharp decline after the inflection point of the curve indicates aggregation of the investigated protein. The black line depicts the heat signals while red line is the single peak fit to the observed unfolding. (TIF) [file pone.0127661.s003.tif]
