# Supplementary material for: N-Terminal Region of the Catalytic Domain of Human N-Myristoyltransferase 1 Acts as an Inhibitory Module
Source: PLoS One. 2015 May 22;10(5):e0127661. doi: 10.1371/journal.pone.0127661 (PMC4441422; doi:10.1371/journal.pone.0127661)
Supplement: S1 Table — (DOC) [file pone.0127661.s004.doc]

**Table S1. List of plasmids used in the study and their characteristics.**

| **Plasmid** | **Encoded Proteins** | **Selection Marker** | **Source** |
| --- | --- | --- | --- |
| pETDuet-1Δ6His_Nef | Nef | Amp*^r^* |  |
| pETDuet-1Δ6His_hNMT_Nef | NMT1^a^; Nef | Amp*^r^* |  |
| pETDuet-1Δ6His_ Δ28-hNMT_Nef | NMT1 ^b^; Nef | Amp*^r^* | This study |
| p27-hNMT1s | NMT1 ^a^ | Kan*^r^* |  |
| p27-Δ28-hNMT1s | NMT1^b^ | Kan*^r^* | This study |

**^a^** Encodes full-length catalytic domain of hNMT1 encompassing. 416 amino-acids. **^b^** Encodes catalytic domain of hNMT1 having N-terminal deletion of 28 amino-acids.
